# Variants in clopidogrel-relevant genes and early neurological deterioration in ischemic stroke patients receiving clopidogrel

**DOI:** 10.1186/s12883-020-01703-6

**Published:** 2020-04-28

**Authors:** Xingyang Yi, Qiang Zhou, Yongyin Zhang, Ju Zhou, Jing Lin

**Affiliations:** 1 Department of Neurology, the People’s Hospital of Deyang City, Deyang, 618000 Sichuan China; 2 Department of Neurology, the Third Affiliated Hospital of Wenzhou Medical University, 108 Wanson Road, Ruian City, Wenzhou, 325200 Zhejiang China

**Keywords:** Ischemic stroke, Early neurological deterioration, Clopidogrel, Genetic polymorphism, Platelet membrane receptor, Glycoprotein IIIa, Pharmacogenetics

## Abstract

**Background:**

Early neurological deterioration (END) is common in acute ischemic stroke (IS). However, the underlying mechanisms for END are unclear. The aim of this study was to evaluate the associations of 16 variants in clopidogrel-relevant genes and interactions among these variants with END in acute IS patients receiving clopidogrel treatment.

**Methods:**

We consecutively enrolled 375 acute IS patients between June 2014 and January 2015. Platelet aggregation was measured on admission and after the 7–10 days of clopidogrel treatment. The 16 variants in clopidogrel-relevant genes were examined using mass spectrometry. The primary outcome was END within the 10 days of admission. Gene-gene interactions were analyzed by generalized multifactor dimensionality reduction (GMDR) methods.

**Results:**

Among the 375 patients, 95 (25.3%) patients developed END within the first 10 days of admission. Among the 16 variants*,* only CYP2C19*2 (rs4244285) AA/AG was associated with END using single-locus analytical approach. GMDR analysis revealed that there was a synergistic effect of gene-gene interactions among CYP2C19*2 rs4244285, *P2Y12* rs16863323, and *GPIIIa* rs2317676 on the risk for END. The high-risk interactions among the three variants were associated with the higher platelet aggregation and independent predictor for END after adjusting for the covariates (hazard ratio: 2.82; 95% confidence interval: 1.36–7.76; *P =* 0.003).

**Conclusions:**

END is very common in patients with acute IS. The mechanisms leading to END are most likely multifactorial. Interactions among CYP2C19*2 rs4244285, *P2Y12* rs16863323, and *GPIIIa* rs2317676 may confer a higher risk for END. It was very important to modify clopidogrel therapy for the patients carrying the high-risk interactive genotypes.

**Clinical trial registration information:**

The study described here is registered at http://www.chictr.org/ (unique Identifier: ChiCTR-OCH-14004724). The date of trial registration was May 30, 2014.

## Background

Stroke is one of the leading causes of adult death and disability [1, 2]. The incidence of early neurological deterioration (END) is very high in patients with an acute ischemic stroke (IS) [3], and END is associated with increased mortality and morbidity [3–6]. It is very vital to understand the mechanisms of END for preventing END in clinic. Although various factors are associated with END risk [7, 8], the underlying mechanisms of END are not fully clear.

Platelet activation plays a vital role in the pathophysiology of IS [9, 10]. Antiplatelet treatment is recommended after IS [11]. Compared with aspirin, clopidogrel was more effective in reducing the vascular events in patients with atherosclerotic disease [12]. However, a proportion of patients receiving clopidogrel have clopidogrel resistance (CR) [13]. The IS patients with CR have an increased risk of recurrent ischemic stroke (RIS) or other vascular events [14–16].

Clopidogrel requires intestinal absorption, metabolic activation in liver, blocks adenosine diphosphate (ADP) binding to platelet P2Y12 receptor in platelet, prevents activation of glycoprotein GPIIb/IIIa complex, which is required for platelet activation [17]. Clopidogrel sensitivity or CR was affected by genetic polymorphisms of clopidogrel absorption, metabolic activation, P2Y12 receptor*,* and glycoprotein IIIa [16, 18]. Previous results have shown that variants of these clopidogrel-relevant genes and these interactions are independently associated with CR and RIS in IS patients receiving clopidogrel [16, 19–21]. Our recent studies revealed that CR and CYP2C19*2 reduced-function alleles were associated with the higher risk for END, and dual antiplatelet therapy could reduce the risk for END in IS patients carrying CYP2C19*2 reduced-function alleles [22, 23]. However, the association between other variants in clopidogrel-relevant genes and END remains to be determined.

Therefore, we hypothesized that clopidogrel-relevant genetic variants and interactions among these variants might influence platelet activation, and were associated with the higher risk for END in acute IS patients receiving clopidogrel. To test this hypothesis, we evaluated 16 variants in clopidogrel-relevant genes and platelet aggregation in 375 acute IS patients on the basis of our previous data [19, 22, 23]. This study was expected to provide new insight into the mechanisms for END and better prevent or treat END.

## Methods

### Study populations

This prospective, observational study was conducted in the People’s Hospital of Deyang City and the Third Affiliated Hospital of Wenzhou Medical University. The protocol of study was reviewed and approved by the Ethics Committees of participating hospitals. Before enrollment into the study, the written informed consent was obtained from each of participants or their family members.

The detailed procedures for recruitment of patients, inclusion criteria, and exclusion criteria were described in our previous studies [19, 22, 23]. In brief, a total of 375 IS patients with first-time stroke and within 72 h of their stroke onset were consecutively enrolled between June 2014 and January 2015. All IS patients were atherothrombosis or small artery disease stroke, National Institutes of Health Stroke Scale (NIHSS) scores were less than 15 points. All patients received standard therapies based on guideline [11], including clopidogrel 75 mg once daily. Various risk factors were recorded. Hyperlipidemia was defined as triglycerides > 180 mg/dL, total plasma cholesterol > 200 mg/dL, or use of lipid-lowering drugs.

### Platelet aggregation

On admission and after 7–10 days of clopidogrel, platelet aggregation was evaluated by light transmittance aggregometry. The results were presented as the amplitudes of light transmittance at 5 min after addition of 10 μM ADP or 0.5 mM arachidonic acid (AA) (Helena Laboratories, Beaumont, TX, USA). The detailed procedure was described in our previous studies [22, 23].

#### Genotyping

The 16 single nucleotide polymorphisms (SNPs) in clopidogrel-relevant genes, including *ABCB1* (rs2032562), *CYP3A4* (rs2242480), *CYP3A5* (rs776746), *CYP2C9* (rs1057910, rs1799853), *CYP2C8* (rs1934980, rs17110453), *CYP2C19* (rs4986893, rs4244285), *P2Y1* (rs1371097, rs1439010, rs701265), *P2Y12* (rs9859538, rs16863323), and *GPIIIa* (rs11871251, rs2317676) were selected from NCBI database (http://www.ncbi.nlm.nih.gov/SNP), according to the below criteria: (1) the SNPs had been assessed in previous studies [16, 19–21]; (2) the SNPs can lead amino acid changes; (3) Tagging SNPs may across diverse human populations (http://pga.gs.washington.edu). We used matrix-assisted laser desorption/ionization time of flight mass spectrometry method to evaluated genotypes of the 16 variants, as our previously described [19, 21].

### Clinical outcomes

The primary outcome was END, which was defined as an increase in total NIHSS score ≥ 2 points during the first 10 days of admission, while excluding new infarct in other vascular territory or hemorrhagic transformation (HT) of infarct or intracerebral hemorrhage (ICH) [3, 22, 23]. The secondary outcome was a composite of MI, RIS, and death within the first 10 days of admission. Safety outcomes were hemorrhagic episodes, including asymptomatic or symptomatic ICH, symptomatic or asymptomatic HT, and extracranial hemorrhages. The investigators who assessed outcomes were blinded to the results of genotypes.

### Statistical analysis

The samples were calculated according to suggested sample requirement of gene-gene interactions [24]. With a two-sided type I error of 0.05, supposing an END event rate of 20% in patients carrying low-risk interactive genotypes, we calculated a sample of 370 could provide 80% power to discover a relative risk increment of 10% for END in patients carrying high-risk interactive genotypes.

All statistical analyses were performed by SPSS 16.0 (SPSS Inc., Chicago, IL, USA). Continuous variables were compared using the Student’s t-test. Discrete variables were assessed using the χ^2^ test. The χ^2^ test was also used to analyze the Hardy-Weinberg equilibrium of genotype frequencies and differences of genotypes in 16 variants between patients with and without END. Generalized multifactor dimensionality reduction method (GMDR, β version 0.7, www.healthsystem.virginia.edu/internet/addiction-genomics/Software) was used to investigate the gene-gene interactions among the 16 variants [19, 21, 25].

Differences in END, other outcomes, and platelet aggregation between the patients with or without high-risk interactive genotypes were compared by χ^2^-test. Survival function for END was assessed by Kaplan-Meier analysis. Survival curves were truncated at 10 days, log-rank test was used to determine the differences between patients with and without high-risk interactive genotypes. The risk for END was evaluated using Cox proportional-hazards model, and was reported as hazard ratio (HR) and 95% confidence interval (CI). Variables entered Cox proportional-hazards model to adjust were the variables of significant association with END (*P* < 0.05) in univariate analysis. All tests were two-sided, A *P* value of less than 0.05 was defined as statistically significant.

## Results

### Incidence of END

Among the 375 enrolled patients, 95 (25.3%) patients suffered from END during the first 10 days of admission. Baseline characteristics and platelet aggregation in patients were summarized in Table 1 and in Table 2 in our previous articles [22, 23]. Briefly, the old age, diabetes mellitus, hemoglobin A1c, fasting glucose, platelet aggregation induced by AA or ADP on admission and after 7–10 days of treatment were associated with END in univariate analysis [22, 23].

**Table 1:** Allelic frequencies of SNPs in patients with and without END

	Patients with END (*n* = 95)	Patients without END (*n* = 280)	*p* value
*CYP2C8* (rs17110453)			
AA	43 (45.3)	132 (47.1)	0.751
AC + CC	52 (54.7)	148 (52.9)	
*CYP2C8* (rs1934980)			
CC	15 (15.8)	40 (14.3)	0. 723
CT + TT	80 (84.2)	240 (85.7)	
*CYP2C9* (rs1799853)			
CC	95 (100)	280 (100)	–
*CYP2C9* (rs1057910)			
AA	85 (89.5)	245 (87.5)	0.626
AC + CC	10 (10.5)	35 (12.5)	
*CYP3A4*(rs2242480)			
CC	51 (53.7)	155 (55.4)	0.786
TT + CT	44 (46.3)	125 (44.6)	
*ABCB1*(rs2032562)			
GG	25 (26.3)	65 (23.2)	0.532
TT + GT	70 (73.7)	215 (76.8)	
*P2Y1*(rs701265)			
AA	46 (48.4)	158 (56.4)	0.193
AG + GG	49 (51.6)	122 (43.6)	
*P2Y1*(rs1439010)			
AA	48 (50.5)	156 (55.7)	0.402
AG + GG	47 (49.5)	124 (44.3)	
*P2Y1*(rs1371097)			
CC	45 (47.4)	161 (57.5)	0.096
TT + CT	50 (52.6)	119 (42.5)	
*CYP3A5*(rs776746)			
AA	12 (12.6)	36 (12.9)	0.998
GG + AG	83 (87.4)	244 (87.1)	
*CYP2C19*(rs4244285)			
GG	20 (16.8)	133 (47.5)	< 0.001
AG + AA	75 (78.9)	147 (52.5)	
*CYP2C19*(rs4986893)			
GG	86 (90.5)	262 (93.6)	0.752
AG	9 (9.5)	18 (6.4)	
*P2Y12*(rs16863323)			
CC	16 (16.8)	62 (22.1)	0.276
TT + CT	79 (83.2)	218 (77.9)	
*P2Y12*(rs9859538)			
GG	68 (71.6)	206 (73.6)	0.705
AG + AA	27 (28.4)	74 (26.4)	
*GPIIIa* (rs2317676)			
AA	62 (65.3)	188 (67.1)	0.724
AG + GG	33 (34.7)	92 (32.9)	
*GPIIIa* (rs11871251)			
AA	27 (28.4)	86 (30.7)	0.678
AG + GG	68 (71.6)	194 (69.3)	

*SNPs* single nucleotide polymorphisms; END, early neurological deterioration

**Table 2:** Comparison of the best models, prediction accuracies, cross-validation consistencies, and *P* values for END identified by generalized multifactor dimensionality reduction analysis

Best model^a^	Training balanced accuracy	Testing balanced accuracy	Cross-validation consistency	Sign test (*P* value)
16	0.613	0.582	7/10	6 (0.432)
1,2	0.418	0.534	8/10	8 (0.296)
1, 2, 3	0.612	0.623	10/10	9 (0.019)
1, 2, 3, 4	0.513	0.586	8/10	6 (0.632)
1, 2, 3, 4, 5	0.617	0.522	9/10	8 (0.763)
1, 2, 3, 4, 5, 6	0.543	0.514	7/10	7 (0.604)
1, 2, 3, 4, 5, 6, 7	0.476	0.495	6/10	5 (0.835)
1, 2, 3, 4, 5, 6, 7, 8	0.543	0.566	7/10	7 (0.351)
1, 2, 3, 4, 5, 6, 7, 8, 9	0.446	0.552	8/10	5 (0.675)
1, 2, 3, 4, 5, 6, 7, 8, 9, 10	0.612	0.624	6/10	6 (0.723)
1, 2, 3, 4, 5, 6, 7, 8, 9, 10,11	0.575	0.513	7/10	7 (0.372)
1, 2, 3, 4, 5, 6, 7, 8, 9, 10,11,12	0.484	0.497	5/10	6 (0.667)
1, 2,3, 4, 5, 6, 7, 8, 9, 10,11,12,13	0.597	0.583	7/10	5 (0.432)
1, 2, 3, 4, 5, 6, 7, 8, 9, 10,11,12,13,14	0.582	0.605	8/10	6 (0.317)
1, 2, 3, 4, 5, 6, 7, 8, 9, 10,11,12,13,14,15	0.622	0.572	6/10	8 (0.186)
1, 2, 3, 4, 5, 6, 7, 8, 9, 10,11,12,13,14,15,16	0.475	0.402	4/10	5 (0.726)

*END* early neurological deterioration

^a^ Numbers 1–16 represent rs4244285, rs2317676, rs16863323, rs17110453, rs11871251, rs776746, rs1371097, rs701265, rs1439010, rs2242480, rs9859538, rs4986893, rs1934980, rs1799853, rs1057910, and rs2032562, respectively

### Association of SNPs in clopidogrel-relevant genes with END

The 16 variants examined were in Hardy-Weinberg equilibrium (*P* > 0.05). The frequency of CYP2C19*2 AA/AG (reduced-function alleles) was higher in patients with END compared with those without END (*P* < 0.001, Table 1). The allelic frequencies of the other 15 variants did not differ between the two groups using single-locus analytical method (*P* > 0.05, Table 1).

### Gene-gene interactions and END

Then, GMDR method was used to investigate the associations of high-order interactions of these SNPs with END, and results showed that there was a significant gene-gene interaction among the 16 variants. The best model for END was CYP2C19*2 rs4244285, *P2Y12* rs16863323, and *GPIIIa* rs2317676 after adjusting covariates, which scored 9 out of 10 for the sign test and 10 out of 10 for cross-validation consistency (*P* = 0.019, Table 2). The one-locus model was computed for each variant. The significance of gene-gene interaction was further determined using a permutation test (*P* = 0.025). These indicate that synergistic action of the three variants significantly contributed to END.

Furthermore, we evaluated the associations of varied genotype combinations of rs4244285, rs16863323 and rs2317676 with END. Compared to patients carrying wild-type genotypes rs4244285GG, rs16863323CC, and rs2317676AA, the relative risk of END for different genotype combination of the three variants was analyzed. Three genotype combinations making larger contribution to END were those patients carrying rs4244285AA, rs2317676GG, and rs16863323TT (OR = 2.72, 95% CI: 1.16–6.76, *P* = 0.007); rs4244285AA, rs2317676AG/GG, and rs16863323TT (OR = 1.93, 95% CI: 1.04–4.12, *P* = 0.037); rs4244285AA, rs2317676AG, and rs16863323CT (OR = 2.38, 95%CI: 1.12–5.54, *P* = 0.026) (Table 3). The three combinations of rs4244285, rs16863323 and rs2317676 were considered as the high-risk interactive genotypes, and the other combinations of the three variants were considered as the low-risk genotypes (Table 3).

**Table 3:** Associations between genotype combinations and END

rs4244285	GG	AA	AA	AA	AG	AA, AG	AA	AA, AG
rs16863323	CC	TT	TT	CT	CT	TT	TT, CT	TT, CT
rs2317676	AA	GG	AG, GG	AG	AG	GG	GG	GG, AG
OR	1 ^a^	2.72	1.93	2.38	1.32	1.14	1.05	1.07
95% CI	–	1.16–6.76	1.04–4.12	1.12–5.54	0.68–2.12	0.72–1.87	0.86–2.53	0.66–1.95
P value	–	0.007	0.037	0.026	0.434	0.325	0.482	0.583

*END* early neurological deterioration, *OR* odds ratio, *CI* confidence interval

^a^The low-risk genotype for each genetic factor was used as the reference

### High-risk interactive genotypes, platelet aggregation, and clinical outcomes

Platelet aggregation and inhibition of platelet aggregation induced by AA or ADP did not significantly differ among the 16 variants (all *P* > 0.05). However, the platelet aggregation on admission and after 7–10 days of clopidogrel was higher, and inhibition of platelet aggregation was lower in patients carrying high-risk interactive genotypes compared to those patients carrying low-risk interactive genotypes (Table 4).

**Table 4:** Association of high-risk interactive genotypes with platelet aggregation and clinical outcomes

	patients carrying the high-risk interactive genotypes (*n* = 87)	patients carrying the low-risk interactive genotypes (*n* = 288)	*p* value
AA-induced platelet aggregation (%)			
before clopidogrel	85.3 ± 14.8	71.2 ± 17.4	< 0.001
after 7–10 days clopidogrel	55.5 ± 12.6	35.3 ± 14.9	< 0.001
inhibition	30.2 ± 9.2	38.1 ± 11.4	< 0.001
ADP-inducedplatelet aggregation (%)			
Before clopidogrel	79.1 ± 16.2	67.9 ± 14.8	< 0.001
after 7–10 days clopidogrel	45.2 ± 13.2	25.6 ± 8.6	< 0.001
inhibition	34.9 ± 12.3	46.4 ± 13.6	< 0.001
END (*n*, %)	34 (39.1)	61 (21.2)	< 0.001
RIS (*n*, %)	1 (1.1)	2 (0.7)	0.963
MI (*n*, %)	0 (0.0)	1 (0.3)	0.586
Death (*n*, %)	1 (1.1)	1 (0.3)	0.754
Safety outcomes			
Asymptomatic HT (n, %)	1 (1.1)	6 (2.1)	0.438
Asymptomatic ICH (n, %)	1 (1.1)	1 (0.3)	0.754
Extracranial bleeding (n, %)	2 (2.3)	9 (3.1)	0.443

*AA* arachidonic acid, *ADP* adenosine diphosphate, *END* early neurological deterioration, *MI* myocardial infarction, *RIS* recurrent ischemic stroke, *HT* hemorrhagic transformation, *ICH* intracerebral hemorrhage

The incidence of END was significantly higher in patients carrying high-risk interactive genotypes than those patients carrying low-risk interactive genotypes (Table 4). There was no significant difference in second outcome and safety outcome between the two groups (Table 4).

### Cox proportional-hazards model analysis for END

**Fig. 1:**
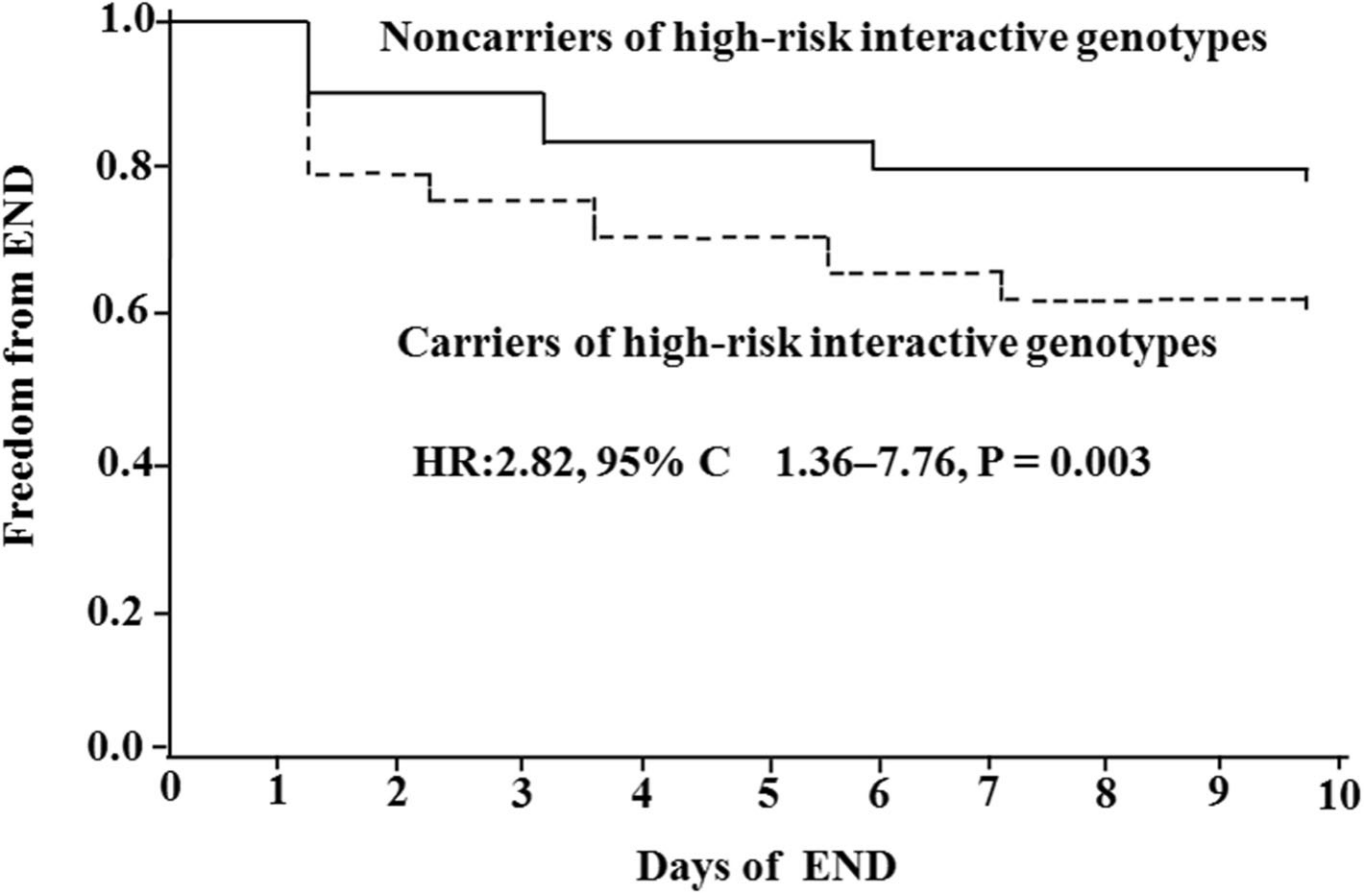
Probability of Survival Free of END. Kaplan-Maier analysis of cumulative freedom from END associated with high-risk interactive genotype (**Figure**). END indicates early neurological deterioration

The risk conferred by the combinations of rs4244285, rs16863323 and rs2317676 was defined as interactive variable. The high-risk interactive genotypes were considered as one, and the low-risk interactive genotypes were considered as zero. Cox regression analysis showed that the high-risk interactive variable was independent predictor for END after adjusting for the covariates (HR: 2.82; 95% CI: 1.36–7.76; *P =* 0.003) (Table 5). Cumulative freedom from END was lower in patients carrying high-risk interactive genotypes than those carrying low-risk interactive genotypes (Fig. 1).

**Table 5:** Cox regression analysis of risk factors for END

Factor	HR	95% CI	*P* value
Age	0.82	0.59–1.28	0.476
Diabetes mellitus	1.67	0.92–2.13	0.135
Hemoglobin A1C	1.08	0.88–2.36	0.198
Fasting blood glucose	0.97	0.82–1.86	0.395
AA-induced platelet aggregation	0.73	0.72–1.69	0.512
ADP-induced platelet aggregation	0.78	0.76–1.83	0.438
*CYP2C19*rs4244285AA/AG	2.41	1.27–6.82	0.005
High-risk interactive variable	2.82	1.36–7.76	0.003

*END* early neurological deterioration, *HR* hazard ratio, *CI* confidence interval

HR for continuous variables means per 1-Standard Deviation increase

## Discussion

In this study, we investigated the incidence of END and its association with 16 variants in clopidogrel-relevant genes in acute IS patients. The results revealed that 25.3% of patients suffered from END. There was a synergistic effect of gene-gene interactions among CYP2C19*2 rs4244285, *P2Y12* rs16863323, *GPIIIa* rs2317676 on END risk, the high-risk interaction among the three variants was independent predictor for END.

The mechanisms of END are not fully understood. Thrombus extension is one of causes for END [26]. Platelet activation can promote vessel wall damage and atherogenesis, and plays a vital role in thrombus extension [26, 27]. Our previous studies and this study demonstrated that platelet activation was associated with the higher risk of END [22, 28, 29]. Insufficient inhibition of platelet activation using antiplatelet treatment may lead to thrombus extension or larger thrombus, further may lead to END [5, 30]. Thus, intensive antiplatelet treatment may be adequate for preventing END in the patients with high platelet activation [22, 23].

Previous studies have revealed that carriers of CYP2C19*2 loss-of-function (LOF) allele are associated with CR and higher risk of vascular events than noncarriers in patients receiving clopidogrel [16, 19, 20]. Our current results and previous study revealed that CYP2C19*2 rs4244285AA/AG was independently associated with the risk of END [23]. Thus, it is very important to modify clopidogrel treatment for carriers of CYP2C19*2 LOF allele, such as dual therapy with aspirin and clopidogrel [23, 28, 31] or substitution of clopidogrel with other antiplatelet drugs [32].

Although our previous study has revealed that CYP2C19*2 AG/AA (reduced-function alleles) was associated with the risk of END in patients treated with clopidogrel [23], the association of other clopidogrel-relevant genetic variants with END risk were not investigated. The important finding in present study was that there was a synergistic effect among rs4244285, rs2317676 and rs16863323 on END risk, the high-risk interactive genotypes of the three variants were independently associated with higher risk of END.

The molecular mechanisms of interactions among the three variants increasing END risk are unclear. Clopidogrel is an inactive prodrug, which requires biotransformation to active metabolite by cytochrome P450 (CYP) enzymes. Polymorphisms of *CYP2C19* affected the pharmacokinetic and pharmacodynamic of clopidogrel [18, 22]. Plasma level of clopidogrel-active metabolite and antiplatelet ability of clopidogrel were lower, and risk of stroke and vascular events were higher in carriers of CYP2C19 *2 LOF allele than non-carriers, who were treated with clopidogrel [16, 18–20]. The pharmacogenetic targets of clopidogrel are P2Y12 receptor and its effector (glycoprotein IIb/IIIa, GPIIIa). P2Y12 receptor and GPIIIa are associated with biologic variability of clopidogrel [33, 34]. *GPIIIa* and *P2Y12* SNPs are independent predictor of CR [35]. Rs4244285, rs2317676 and rs16863323 encode CYP enzymes, glycoprotein receptor and platelet membranes receptors, respectively. The reason of the three variants interactions increasing END risk may be that they all participate in modulation pharmacodynamics, pharmacokinetics, and biologic activity of clopidogrel, which are necessary for IS patients to response to clopidogrel. Our current results demonstrated that platelet aggregation on admission and after 7–10 days of clopidogrel was significantly higher, inhibition of platelet aggregation was significantly lower in patients with high-risk interactive genotypes than those carrying low-risk interactive genotypes. Therefore, we speculate that high-risk interactions among rs4244285, rs16863323 and rs2317676 could provide the IS patients with high platelet activation, which may increase END risk. The results indicate that it is very necessary to modify clopidogrel for those patients carrying high-risk interactive genotypes.

Several potential limitations need to be considered in this study. First limitation was limited sample size, two-center, and short follow-up period. Second, although we investigated the association of high-risk interactive genotypes with platelet activation, we did not measure plasma clopidogrel level and its active metabolite level. Third, despite we investigated the known functional variants of clopidogrel-relevant genes, we did not assess some rare functional variants in this study. Thus, further studies should be necessary in the future.

## Conclusion

Incidence of END was very high in acute IS patients receiving clopidogrel. There was a synergistic effect of CYP2C19*2 rs4244285, *GPIIIa* rs2317676, and *P2Y12* rs16863323 on END risk. The high-risk interaction among the three variants was independent predictor for END. It was necessary to modify clopidogrel therapy for the patients carrying high-risk interactive genotypes. Current study was expected to provide new insight into the genetic mechanisms for END, offer theoretical references for drug discovery and gene therapy in future, modify antiplatelet therapy for the patients carrying the high-risk interactive genotypes promptly, and better prevent or treat END.

## Data Availability

The datasets used and/or analysed during the current study are available from the corresponding author on reasonable request.

## Abbreviations

END: Early neurological deterioration
IS: Ischemic stroke
GMDR: Generalized multifactor dimensionality reduction
MI: Myocardial infarction
CR: Clopidogrel resistance
RIS: Recurrent ischemic stroke
NIHSS: National Institutes of Health Stroke Scale
AA: Arachidonic acid
ADP: Adenosine diphosphate
OR: Odds ratio
HR: Hazard ratio
CI: Confidence interval
HT: Hemorrhagic transformation
ICH: Intracerebral hemorrhage
